# Triterpenoids from *Ainsliaea yunnanensis* Franch. and Their Biological Activities

**DOI:** 10.3390/molecules21111481

**Published:** 2016-11-07

**Authors:** Jinjie Li, Bo Zhang, Hailing Liu, Xuan Zhang, Xiaoya Shang, Changqi Zhao

**Affiliations:** 1 Key Laboratory of Cell Proliferation and Regulation Biology, Ministry of Education, College of Life Science, Beijing Normal University, Beijing 100875, China; lijinjie.7785004@163.com (J.L.); liuhailing@bnu.edu.cn (H.L.); zhangxuan1328@163.com (X.Z.); 2 Beijing Key Laboratory of Bioactive Substances and Functional Foods, Beijing Union University, Beijing 100191, China; 18801127628@163.com

**Keywords:** *Ainsliaea yunnanensis*, triterpenoids, NMR, structures, activity

## Abstract

One new pentacyclic triterpenoid, 3β-carboxylicfilic-4(23)-ene (**1**), and three known pentacyclic triterpenoids, adian-5-en-3α-ol (**2**), fernenol (**3**), and fern-7-en-3β-ol (**4**) were isolated from the petroleum ether phase of the ethanolic extract of *Ainsliaea yunnanensis* Franch. Their structures were established by spectroscopic methods including 1-D and 2-D NMR, and MS experiments. Compounds **1**, **2**, **3**, and **4** showed significant selective cytotoxicity against human acute monocytic leukemia cell line (THP-1) with IC_50_ values of 5.12 μM, 1.78 μM, 1.74 μM, and 1.75 μΜ, respectively. Compound **1** also showed an anti-inflammatory effect through the inhibition of the activity of NF-κB by blocking the nuclear translocation of p65.

## 1. Introduction

The genus *Ainsliaea* (Asteraceae family) includes approximately 70 species distributed primarily in Southeastern Asia, 48 of which are indigenous to China [1]. Many *Ainsliaea* species have been widely used in Chinese folk medicine to treat various diseases including coughing and asthma, rheumatism and arthralgia, traumatic injury, blood hemostasis, enteritis dysentery, pharyngolaryngitis, and urinary system and gynecological diseases [2]. Phytochemical studies on *Ainsliaea* species revealed that they contained sesquiterpenes, triterpenes, flavonoids, and phenolic acid compounds [3,4,5,6]. These components have shown various activities including cytotoxic, anti-inflammatory, pancreatic lipase inhibition, antimicrobial, antihemorrhagic, antioxidant, and antiviral activities [1,3,7,8,9].

*Ainsliaea yunnanensis* Franch. is a traditional Chinese herbal medicine named “zhui feng jian”, “yan mai ling”, and “bone arrow” and is mainly distributed in Yunnan, Guizhou and the southwest region of the Sichuan province in China [10]. However, only a few pharmacology studies (only six patents) have reported on its medicinal effects in the clinical treatment of rheumatoid arthritis, gastrointestinal disease, traumatic injury, dispelling pathogenic wind, dredging collaterals, and pain relief [11,12,13,14,15]. Our previous pharmacology studies have revealed that the petroleum ether phase of the ethanolic extract of *A. yunnanensis* exhibited potent cytotoxic and anti-inflammatory activities, and twelve triterpene compounds were isolated from it [16]. The continuing examination of the petroleum ether extract has resulted in the characterization of one new pentacyclic triterpenoid, 3β-carboxylicfilic-4(23)-ene (**1**), and three known pentacyclic triterpenoids, adian-5-en-3α-ol (**2**), fernenol (**3**), and fern-7-en-3β-ol (**4**). All of them showed significant selective cytotoxicity against the human acute monocytic leukemia cell line (THP-1). Compound **1** also showed an anti-inflammatory effect through the inhibition of the activity of NF-κB by blocking the nuclear translocation of p65.

## 2. Results and Discussion

**Figure 1:**
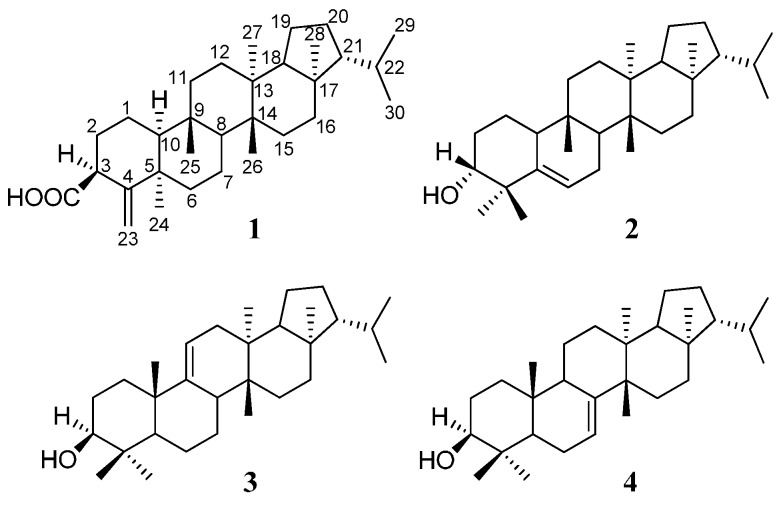
Chemical structures of Compounds **1**, **2**, **3**, and **4**.

The EtOH extract of *A. yunnanensis* was partitioned between water and petroleum ether, EtOAc, and normal butanol. The petroleum ether phase was concentrated under vacuum (<50 °C) and then separated repeatedly by column chromatography over silica gel and Sephadex LH-20 media to obtain compounds **1**, **2**, **3**, and **4** (Figure 1).

**Figure 2:**
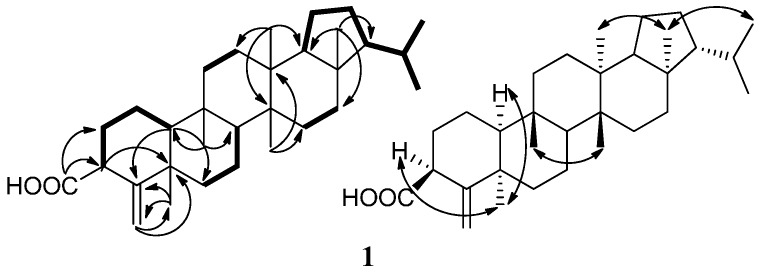
Main ^1^H-^1^H COSY (bold lines), HMBC (arrows) and NOE (double arrows) correlations of compound **1**.

Compound **1** was obtained as a white amorphous powder. The HR-ESIMS at *m*/*z* 453.3728 [M−H]^−^ (calcd. 453.3738) indicated the molecular formula of **1** as C_31_H_50_O_2_. The IR spectrum of **1** suggested that it contained a carboxyl group (1696 cm^−1^) and a double bond (1635 cm^−1^). The ^1^H-NMR spectrum of **1** in C_5_D_5_N displayed signals for five singlet methyl groups (*δ*_H_ 0.71, 0.90, 0.97, 0.98, and 2.02), two doublet methyl groups [*δ*_H_ 0.83 (3H, d, *J* = 6.5 Hz) and 0.88 (3H, d, *J* = 6.5 Hz)], and two olefinic signals (*δ*_H_ 5.06, 5.23). The ^13^C-NMR spectrum showed 31 carbon signals, which were classified as seven methyls, eleven methylenes (one olefinic methylenes), six methines, and seven nonprotonated carbons (one carbonyl and one olefinic carbon) based on the DEPT and HSQC spectra (Table 1). All spectroscopic data above in combination with seven degrees of unsaturation required by the molecular formula suggested that **1** was a pentacyclic triterpene containing one double bond and one carboxyl group. The ^13^C-NMR signals of rings C–E and the isopropyl side chain in **1** were very similar to those of the known compound, 3β-hydroxyfilic-4(23)-ene [17]. However, the signals of rings A and B in **1** and 3β-hydroxyfilic-4(23)-ene are very different. The structure of **1** was finally characterized by the careful analysis of its 2D-NMR spectroscopic data including ^1^H-^1^H COSY and HMBC (Figure 2).

Five structural fragments as shown with bold lines in Figure 2 (C-10 through C-1 to C-3; C-6 through C-7 to C-8; C-11 to C-12; C-15 to C-16; and C-18 through C19 to C-29 and C-30) were first established by the correlations observed in the ^1^H-^1^H COSY spectrum. The connectivity of the five structural fragments, quaternary carbons, and the other functional groups were mainly achieved by the analysis of the HMBC spectrum (Figure 2). Long-range HMBC correlations from H-3 to C-4 and C-5, from H_2_-23 to C-4, C-5, and C-24, and from H_3_-24 to C-4, C-5, and C-23 indicated that olefinic methylene-23 and Me-24 were attached to C-4 and C-5, respectively. Correlations from H-10 to C-4, C-5, and C-6, from H_3_-25 to C-8, C-9, C-10, and C-11, from H_3_-26 to C-8, C-13, C-14, and C-15, from H_3_-27 to C-12, C-13, C-14, and C-18, and from H_3_-28 to C-16, C-17, C-18, and C-21 not only confirmed the presence of a A/B/C/D/E-rings system but also located the Me-25 at C-9, Me-26 at C-14, Me-27 at C-13, and Me-28 at C-17, respectively. In addition, an important correlation from H-2 and H-3 to C-31 combining their chemical shifts suggested that the carboxyl group was attached to C-3. The planar structure of **1** was, therefore, determined as 3-carboxylicfilic-4(23)-ene (Figure 1).

The relative stereochemistry of **1** was elucidated by the analysis of its NOESY data and compared with the known literature [17,18], as shown in Figure 2. NOE correlations between H_3_-24 with H-3 and H-10, and between H_3_-28 with H_3_-27 and H_3_-29 revealed that these protons were cofacial and defined as having an α-orientation, whereas NOE correlations of H_3_-25/H_3_-26 indicated they were in β-orientation. Accordingly, the structure of **1** was established as 3β-carboxylicfilic-4(23)-ene. (The raw data, please see the supplementary materials).

The similar structures were only isolated from *Ainsliaea fragrans* Franch. (fernenol) [19] and *Ainsliaea macrocephala* Franch. (simiarenol) [20], but they were not tested in order to evaluate the possible biological activities.

**Figure 3:**
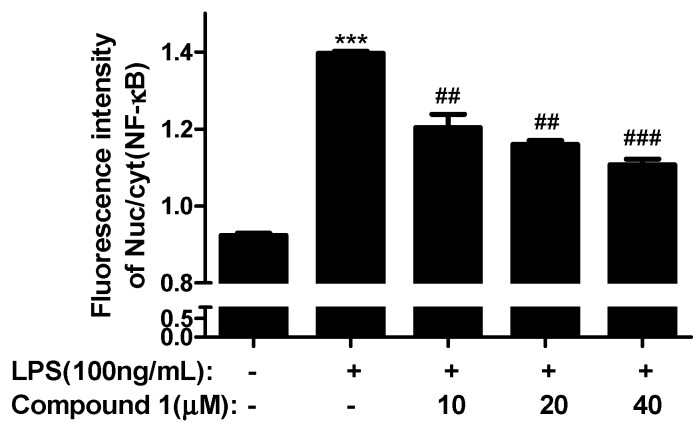
Effect of Compound **1** on the expression of the NF-κB (p65) protein in LPS (100 ng/mL)-induced bone marrow-derived macrophages (BMDM) (Nuc/cyt). *** *p* < 0.001 vs. Control; ^##^ *p* < 0.01, ^###^ *p* < 0.001 vs. LPS.

Compounds **1**, **2**, **3**, and **4** were screened in vitro for their cytotoxicities against five human cell lines including human lung adenocarcinoma (A549), cervical cancer cells (HeLa), epidermoid carcinoma (A431), breast cancer (Mcf-7), and acute monocytic leukemia (THP-1) cell lines and were also evaluated in vitro for their anti-inflammation activity. In cytotoxic activity tests, IC_50_ values of greater than 10 μM were defined as inactive. Compound **1**, **2**, **3**, and **4** showed significant selective cytotoxicity against the THP-1 cell line with IC_50_ values of 5.12 μM, 1.78 μM, 1.74 μM, and 1.75 μM, respectively (Table 2). The screening results of the four compounds against the THP-1 cell line indicated the intra-annular double bond may be a more active group than the exocyclic double bond. Compound **1** also showed an anti-inflammatory effect through the inhibition of the activity of NF-κB by blocking the nuclear translocation of p65. The result was expressed by the fluorescence intensity ratio of the NF-κB subunit p65 expression in the nucleus to that in the cytoplasm (Nuc/cyt). After adding compound **1**, Nuc/cyt was reduced relative to the LPS group (Figure 3).

## 3. Experimental Section

### 3.1. General

IR spectra were recorded as KBr disks on a Nicolet Impact 400 FT-IR Spectrophotometer (Nicolet Instrument. Inc., Madison, WI, USA). One- and two-dimensional NMR spectra were obtained in C_5_D_5_N at 500 MH_Z_ for ^1^H and 125 MH_Z_ for ^13^C, respectively, on a Varian 500 MH_Z_ spectrometer (Bruker Corporation, Billerica, MA, USA) (^1^H, 500.06 MH_Z_; ^13^C, 125.75 MH_Z_) with TMS (HWRK Chem Co., Beijing, China) as a reference. Mass spectra including high-resolution mass spectra were recorded on a JEOL JMS AX-500 spectrometer (JEOL Ltd., Tokyo, Japan). Column chromatography was performed with silica gel (160–200 mesh; Qingdao Marine Chemical Plant, Qingdao, China), RP-18 reverse phase silica gel (43–60 μM), cyanopropyl silica gel (43–60 μM), and Sephadex LH-20 (Pharmacia Biotech AB, Uppsala, Sweden). LPLC separation was performed with Combiflash with a UV detector (ISCO Companion, Lincoln, NE, USA). HPLC separation was performed with a Waters 2545 system with 2998 diode array detector (Waters Corporation, Milford, MA, USA), using a Waters Sunfire (250 mm × 10 mm i.d.) preparative column packed with C18 (5 μM) (Alltech Associates, Inc., Bannockburn, IL, USA). TLC (Qingdao Marine Chemical Plant, Qingdao, China) was carried out with glass precoated silica gel GF_254_ plates. Spots were visualized under UV light and followed by heat spraying with 8% H_2_SO_4_ in 95% EtOH.

### 3.2. Experimental Material

*A. yunnanensis* was obtained from Chuxiong city, Yunan province, China, and was identified by Lu Jin Mei, Associate Researcher at the Kunming Institute of Botany, Chinese Academy of Sciences, Yunnan, China. The specimen has been deposited at Beijing Union University, and the Beijing Key Laboratory of Bioactive Substances and Functional Foods, Beijing, China.

### 3.3. Extraction and Isolation

The dried whole plants of *A. yunnanensis* (10.0 kg) were ground into powder and extracted with 95%, 80%, and 70% aqueous EtOH sequentially, at room temperature for 120 min under sonication. The extract was evaporated under reduced pressure to yield a dark brown residue, which was suspended in H_2_O and then partitioned with petroleum ether, EtOAc, and normal butanol, respectively. The petroleum ether-soluble portion (78.0 g) showing cytotoxic activity (IC_50_ < 50 μg/mL) was fractionated via silica gel column chromatography, eluting with a gradient of increasing acetone (0–100%) in petroleum ether (60–90 °C) to give 10 fractions, sh1–sh10.

The fraction sh6 (2.8 g) was chromatographed over silica gel gradient eluting with petroleum ether–acetone (100:1–10:1) to give seven fractions sh6-1–sh6-7. The fraction sh6-4 was subjected to column chromatography over Sephadex LH-20 gel eluting with petroleum ether–CHCl_3_–CH_3_OH (5:5:1) to five fraction sh6-4-1–sh6-4-5. The sh6-4-4 fraction was heated and dissolved in chloroform. Upon cooling, the white precipitate was filtered to yield compound **1** (12.0 mg). The sh6-3 fraction was purified by Sephadex LH-20 gel, and the mobile phase was petroleum ether–CHCl_3_–CH_3_OH (5:5:1) to obtain five subfractions sh6-3-1–sh6-3-5. Next, we repeatedly purified the sh6-3-4 fraction, which was further purified by LPLC over normal phase cyanopropyl silica, eluting with petroleum ether (60–90 °C) and preparative reversed phase (C18) HPLC, eluting with acetonitrile-H_2_O (98:2, 18.0 mL/min), and a 206 nm detection wavelength was used to collect picks of 18 min, 20 min, and 23 min respectively to yield compound **2** (7.9 mg), compound **3** (7.8 mg), and compound **4** (10.6 mg).

### 3.4. Cytotoxic Activity Assays

Human lung adenocarcinoma (A549), cervical cancer cells (HeLa), epidermoid carcinoma (A431), breast cancer (Mcf-7), and acute monocytic leukemia (THP-1) cell lines were obtained from the American Type Culture Collection (ATCC). Cells were respectively maintained in RRMI1640 supplemented with 10% fetal bovine serum. Cultures were incubated at 37 °C in humidified 5% CO_2_. The assay was carried out adopting the MTT method.

All of the cell lines were seeded in 96-well microtiter plates at 2900 cells/100 μL/well. After 24 h, the compounds were added to the cells. After 24 h of drug treatment, we measured the amount of the metabolic formazan crystals to determine the amount of cell viability. MTT assay results were read using a Bio-RAD iMark Microplate reader (iMark/xMark, Bio-Rad, CA, USA) at 570 nm. All compounds were tested in five concentrations and were dissolved in DMSO with a final DMSO concentration of 0.1% in each well. Each concentration of the compounds were tested in three parallel wells. IC_50_ values were calculated using SPSS software (SPSS 21.0, IBM, New York, NY, USA).

### 3.5. Anti-Inflammatory Activity Assays

Bone marrow-derived macrophage (BMDM) cell lines were obtained from ATCC. Cells were maintained in DMEM supplemented with 10% fetal bovine serum. Cultures were incubated at 37 °C in humidified 5% CO_2_. The assay was carried out adopting immunofluorescence to investigate NF-κB (p65) transportation to the nucleus.

The cell line was seeded in 96-well microtiter plates at 18,000 cells/100 μL/well. Overnight, the compounds were added to the cells. After 4 h, LPS (100 ng/mL) was added and stimulated for 30 min. The supernatant was discarded, and the cells were washed one time with PBS. Next, the cells were fixed for 20 min with 4% paraformaldehyde (100 μL/well, the fixative was discarded, and the cells were washed two times with PBS), punched for 15 min on ice with PBS containing 0.1% Triton X-100 (100 μL/well, the supernatant was discarded, and the cells were washed one time with PBS), and closed on the shaking table for 60 min with PBS containing 5% BSA (100 μL/well). After NF-κB (p65) first antibody (1:300, 50 μL/well) was added and hatched for overnight at 4 °C, the cells were washed three times with PBS containing 0.1% Tween-20 (200 μL/well, each time for 5 min). After that, the secondary antibody (1:300, 50 μL/well) was added and hatched for one hour at room temperature, then the cells were washed four times with PBS containing 0.1% Tween-20 (200 μL/well, each time for 5 min). Finally, the cells were detected by using a Cellomics ArrayScan Infinity (Thermo Fisher Scientific) after 4',6-diamidino-2-phenylindole (DAPI) (1:10000) staining for 5 min. All compounds were tested at three concentrations, and each concentration was tested in three parallel wells. The result was expressed by the fluorescence intensity ratio of the NF-κB subunit p65 expression in the nucleus to that in the cytoplasm (Nuc/cyt).

### 3.6. Spectral Data

*3*β*-Carboxylicfilic-4(23)-ene* (**1**) was obtained as white amorphous powder, 12.0 mg; IR $\nu_{\max}^{\mathrm{KBr}}$: 3445, 3125, 2953, 2870, 1696, 1635, 1471, 1454, 1400, 1381, 1275, 1230, 1169, 1032, 1008, 960, 889, 779, 710 cm^−1^. ^1^H-NMR (500 MHz, C_5_D_5_N) and ^13^C-NMR (125 MHz, C_5_D_5_N) spectral data, see Table 1; EI-MS *m*/*z* 454 [M]^+^, 439, 411, 408, 393, 369, 300, 285, 273, 259, 231, 207, 205, 191, 161, 149, 135, 121, 109, 95, 81, 69, 55; HR-ESIMS *m*/*z* 477.3697 [M + Na]^+^ (Caled. 477.3703) and 453.3728 [M − H]^−^ (Caled. 453.3738).

## Figures and Tables

**Table 1:** ^1^H and ^13^C-NMR spectral data for compound **1**.

NO.	1	
	*δ*_H_	*δ*_C_
1	1.64 m, 1.85 m	27.5
2	1.89 m, 2.16 m	29.6
3	2.99 dd (6.0, 11.0)	54.9
4		151.2
5		52.6
6	1.90 m, 2.15 m	23.7
7	1.42 m, 1.65 m	18.8
8	1.56 m	39.7
9		36.1
10	2.20 m	59.1
11	1.42 m	38.7
12	0.96 m, 1.07 m	28.8
13		39.0
14		40.0
15	1.07 m, 1.28 m	28.9
16	1.50 m	35.6
17		43.0
18	1.54 m	52.0
19	1.21 m, 1.32 m	20.2
20	1.42 m, 1.75 m	28.5
21	0.90 m	60.0
22	1.37 m	30.9
23	5.06 s, 5.23 s	110.1
24	2.02 s	20.1
25	0.97 s	20.3
26	0.98 s	15.3
27	0.90 s	15.3
28	0.71 s	16.1
29	0.88 d (6.5)	22.1
30	0.83 d (6.5)	23.0
31		175.8

^1^H and ^13^H data were measured in C_5_D_5_N for **1** at 500 and 125 MHz. The assignments were based on the DEPT, ^1^H-^1^H COSY, HMQC, and HMBC experiments.

**Table 2:** Cytotoxic data for compounds **1**, **2**, **3**, and **4**.

Compound	IC_50_ (μM)				
	A549	HeLa	A431	Mcf-1	Thp-1
**1**	>10	>10	>10	>10	5.12
**2**	>10	>10	>10	>10	1.78
**3**	>10	>10	>10	>10	1.74
**4**	>10	>10	>10	>10	1.75
topotecan	1.9	1.7	4.4	3.6	1.2

## Supplementary Materials

The data can be accessed at: http://www.mdpi.com/1420-3049/21/11/1481/s1.
