# Supplementary material for: Triterpenoids from Ainsliaea yunnanensis Franch. and Their Biological Activities
Source: Molecules. 2016 Nov 7;21(11):1481. doi: 10.3390/molecules21111481 (PMC6273999; doi:10.3390/molecules21111481)
Supplement: Supplementary file 1 [file molecules-21-01481-s001.pdf]

Supplementary Materials: Triterpenoids from *Ainsliaea yunnanensis* Franch. and Their Biological Activities

Jinjie Li, Bo Zhang, Hailing Liu, Xuan Zhang, Xiaoya Shang and Changqi Zhao

【Sample2-C31H50O2】[M+Na]$^{+}$ theoretical value: 477.3703 [M-H]$^{-}$ theoretical value: 453.3738

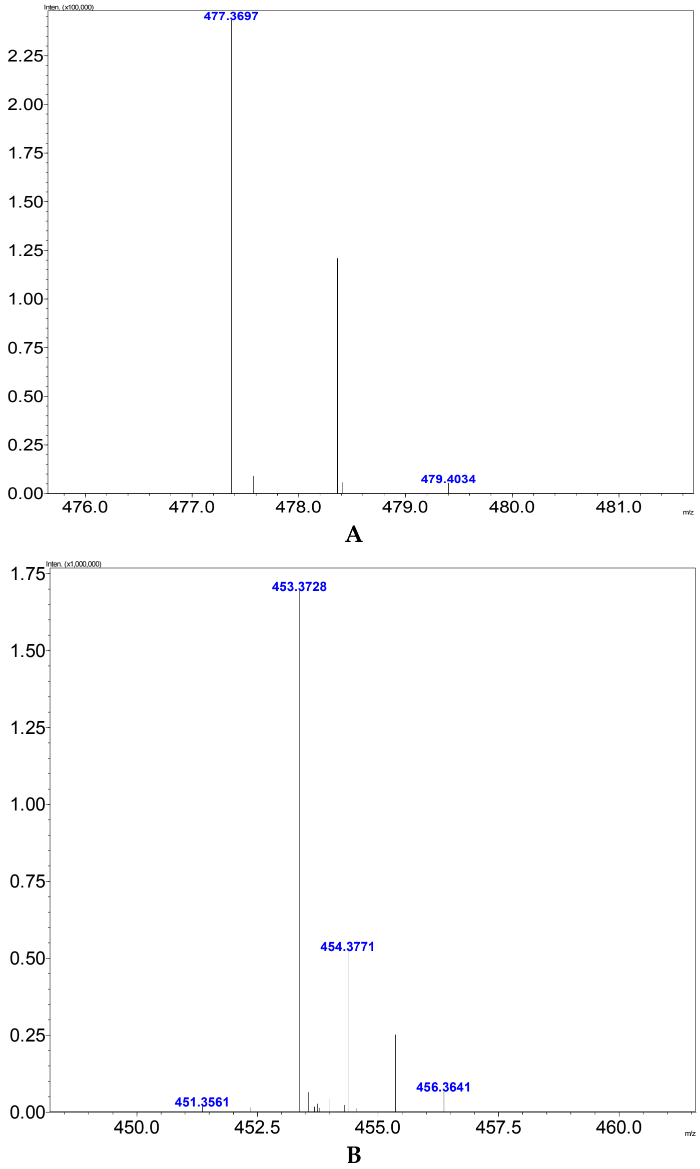

Figure S1. The HRESIMS Spectrum of Compound **1**. (**A**) Positive mode; (**B**) Negative mode.

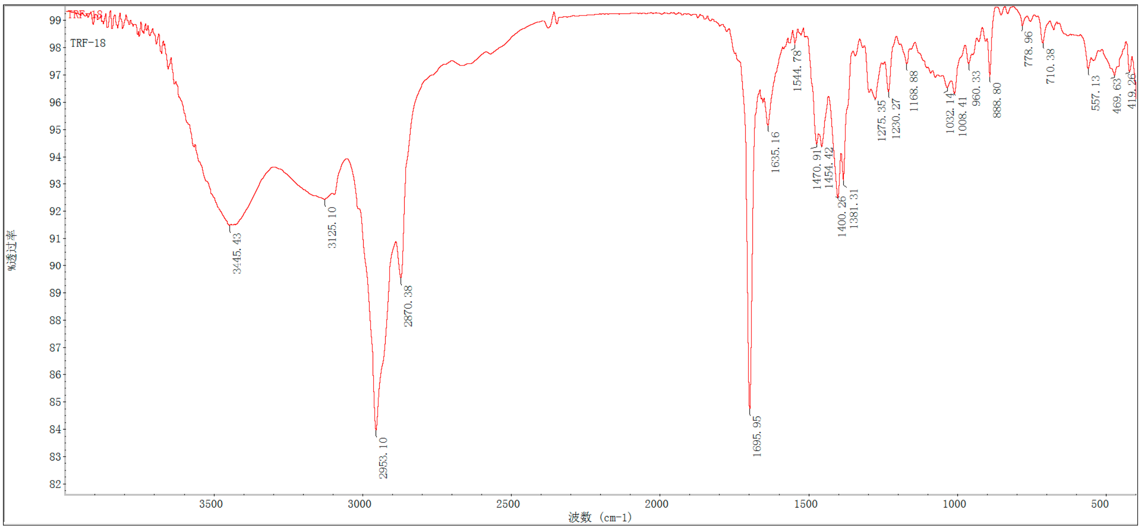

Figure S2. The IR Spectrum of Compound **1**.

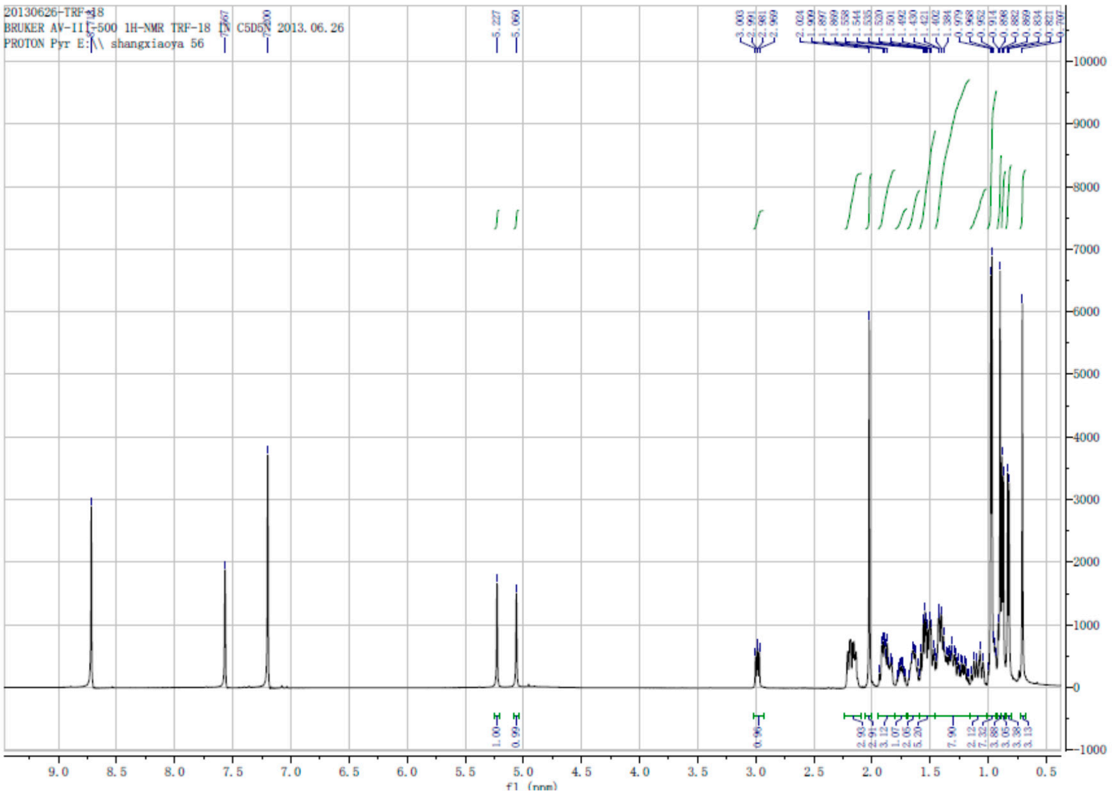

Figure S3. The ^{1}H-NMR Spectrum of Compound **1** in Pyr.

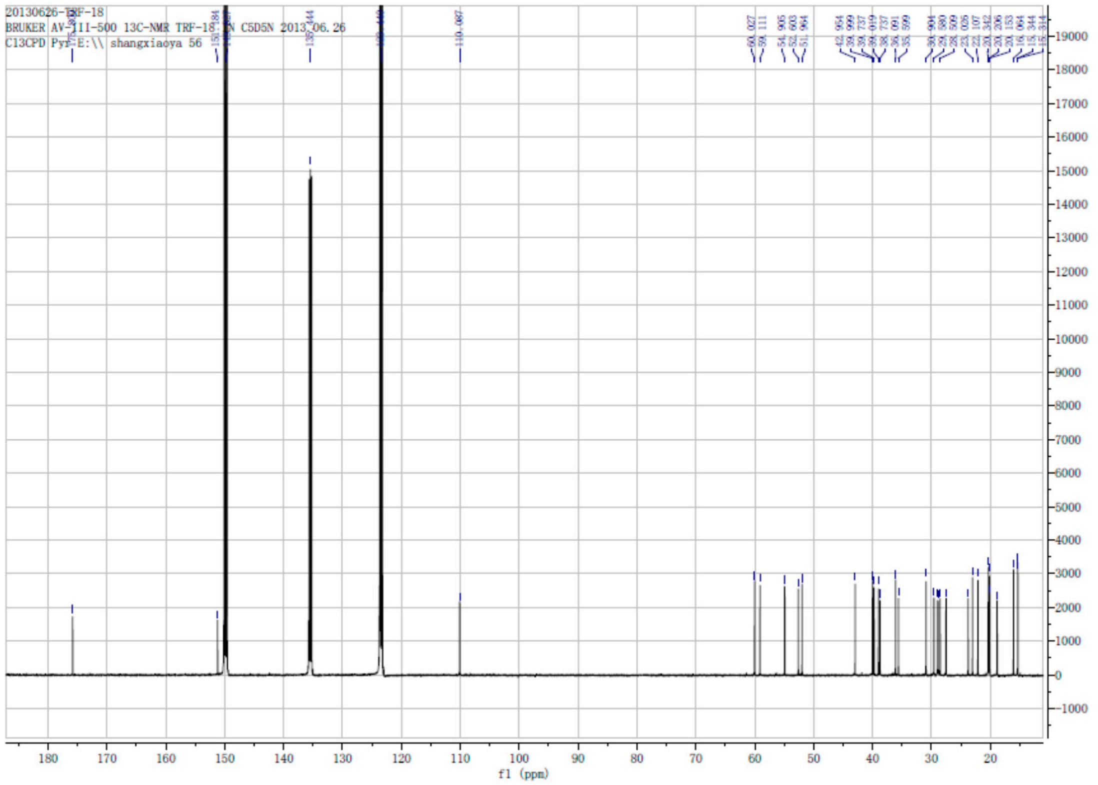

Figure S4. The ^{13}C-NMR Spectrum of Compound **1** in Pyr.

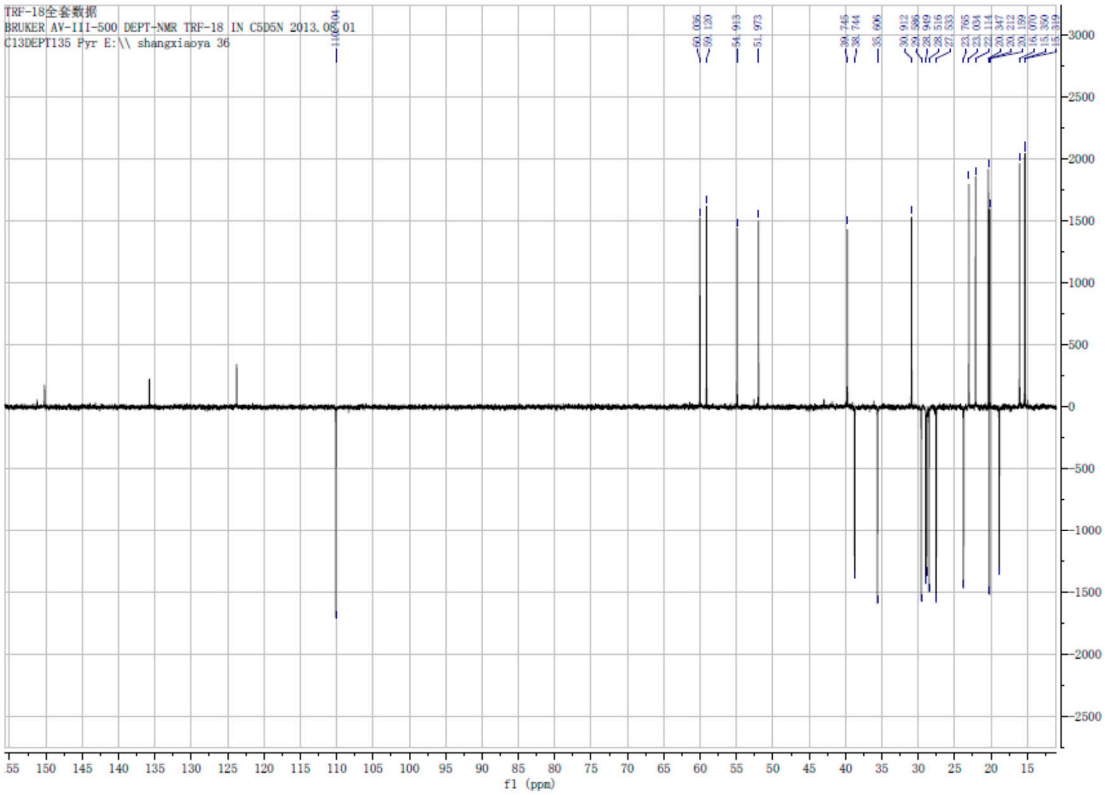

Figure S5. The DEPT 135 Spectrum of Compound **1** in Pyr.

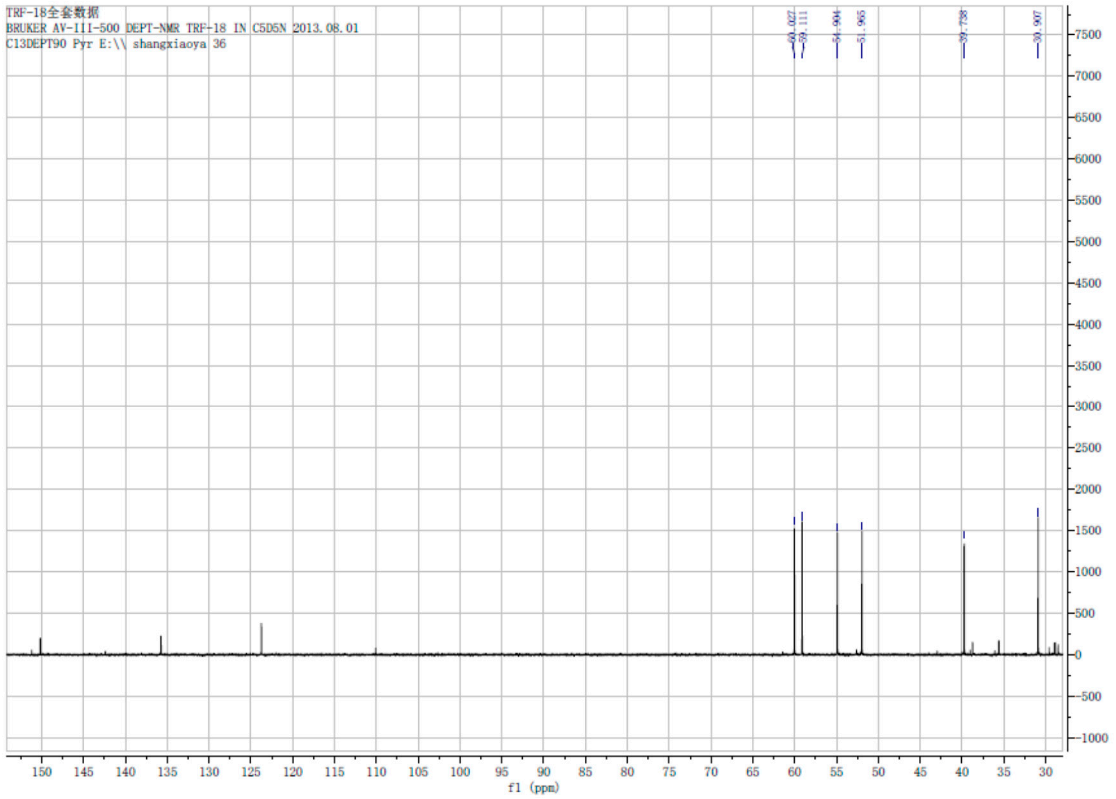

Figure S6. The DEPT 90 Spectrum of Compound **1** in Pyr.

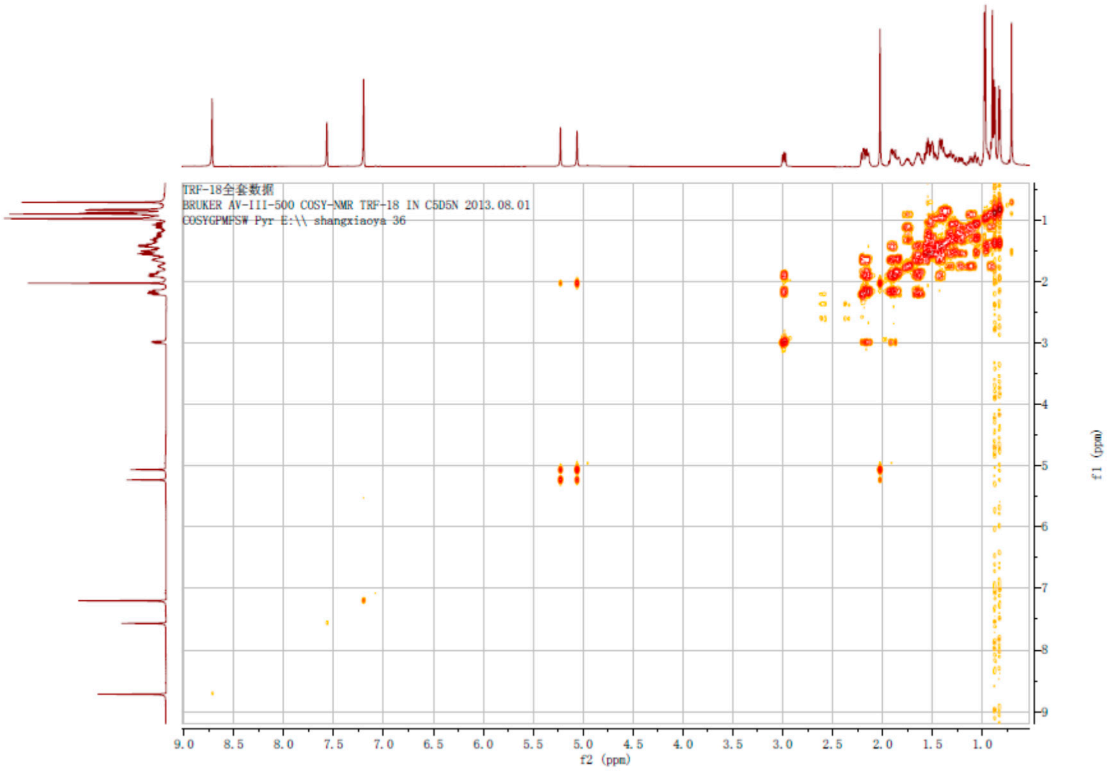

Figure S7. The ^{1}H-^{1}H COSY Spectrum of Compound **1** in Pyr.

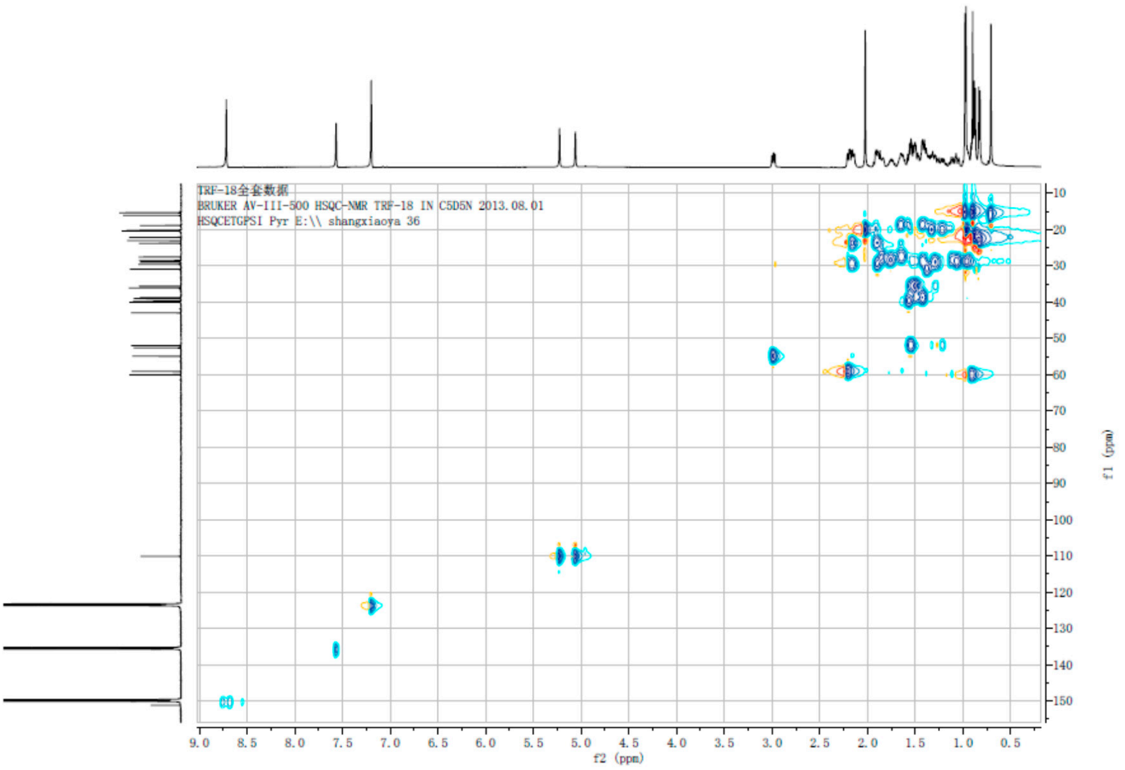

Figure S8. The HSQC Spectrum of Compound **1** in Pyr.

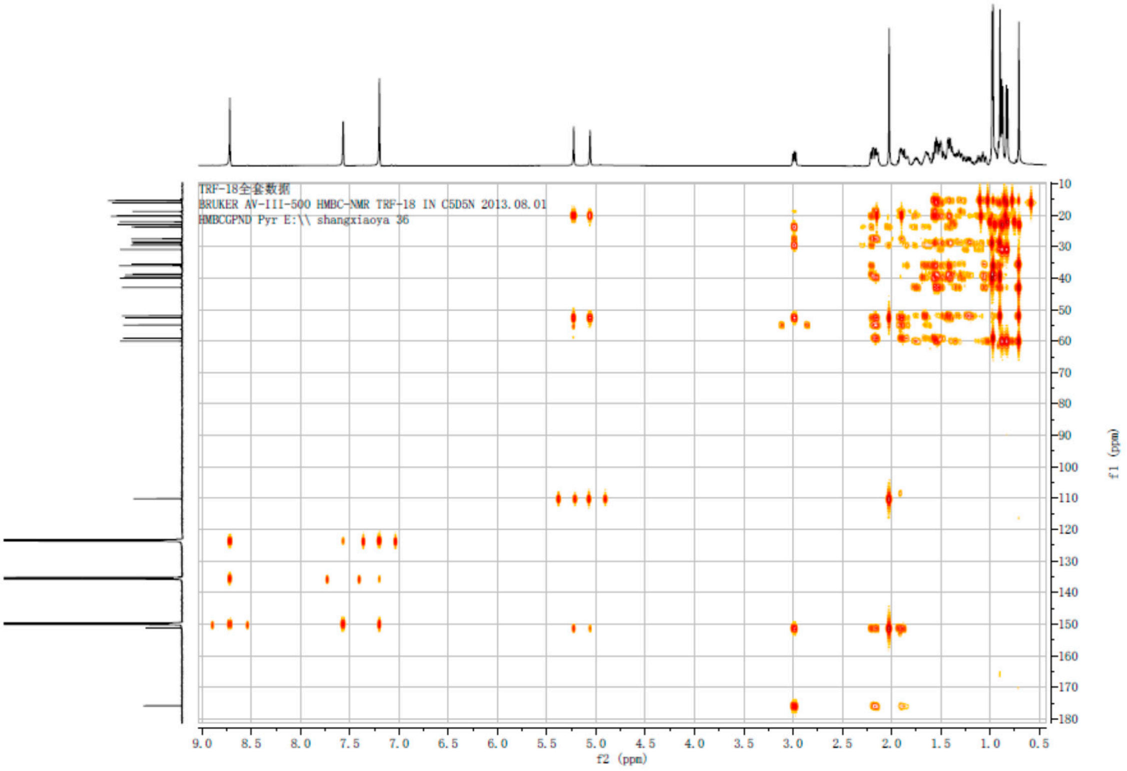

Figure S9. The HMBC Spectrum of Compound **1** in Pyr.

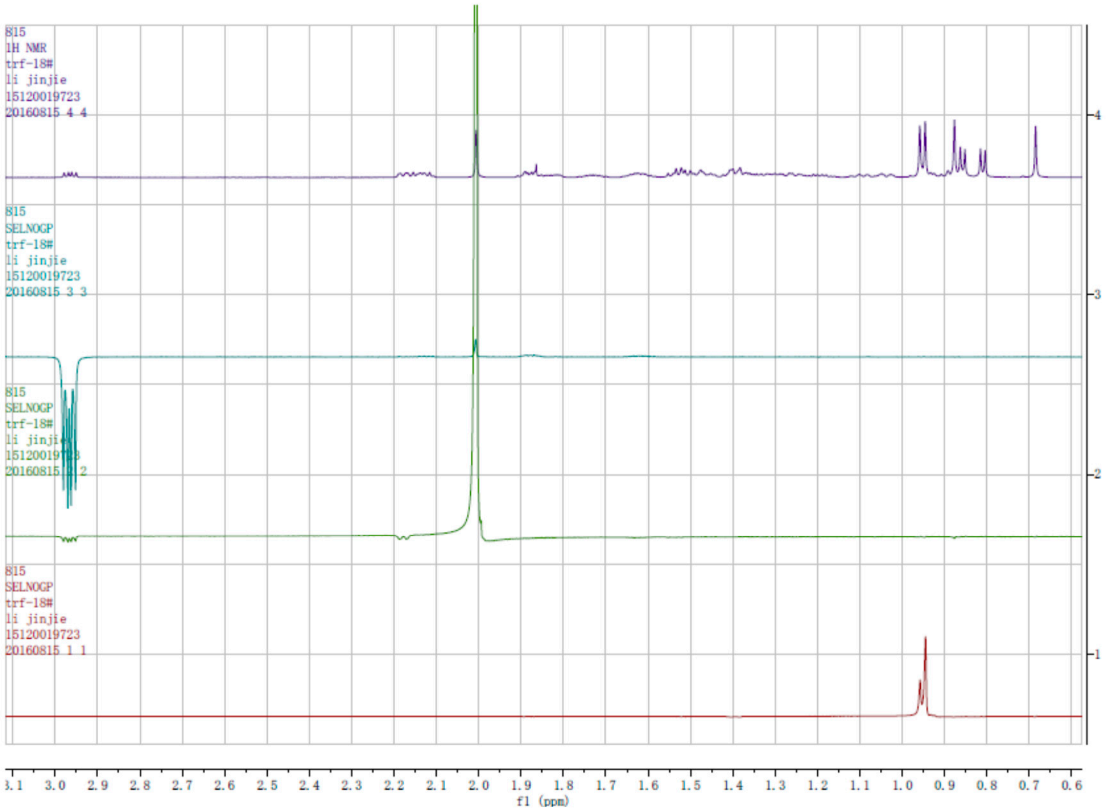

Figure S10. The NOE-1 Spectrum of Compound **1** in Pyr.

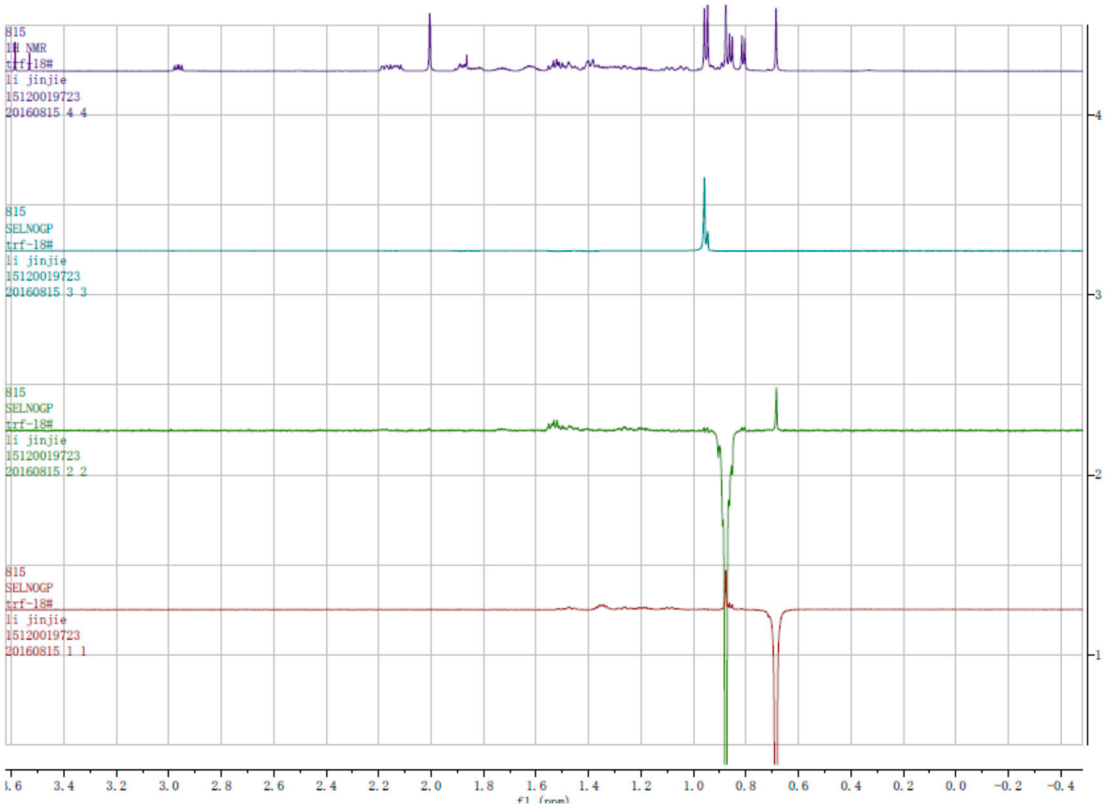

Figure S11. The NOE-2 Spectrum of Compound **1** in Pyr.

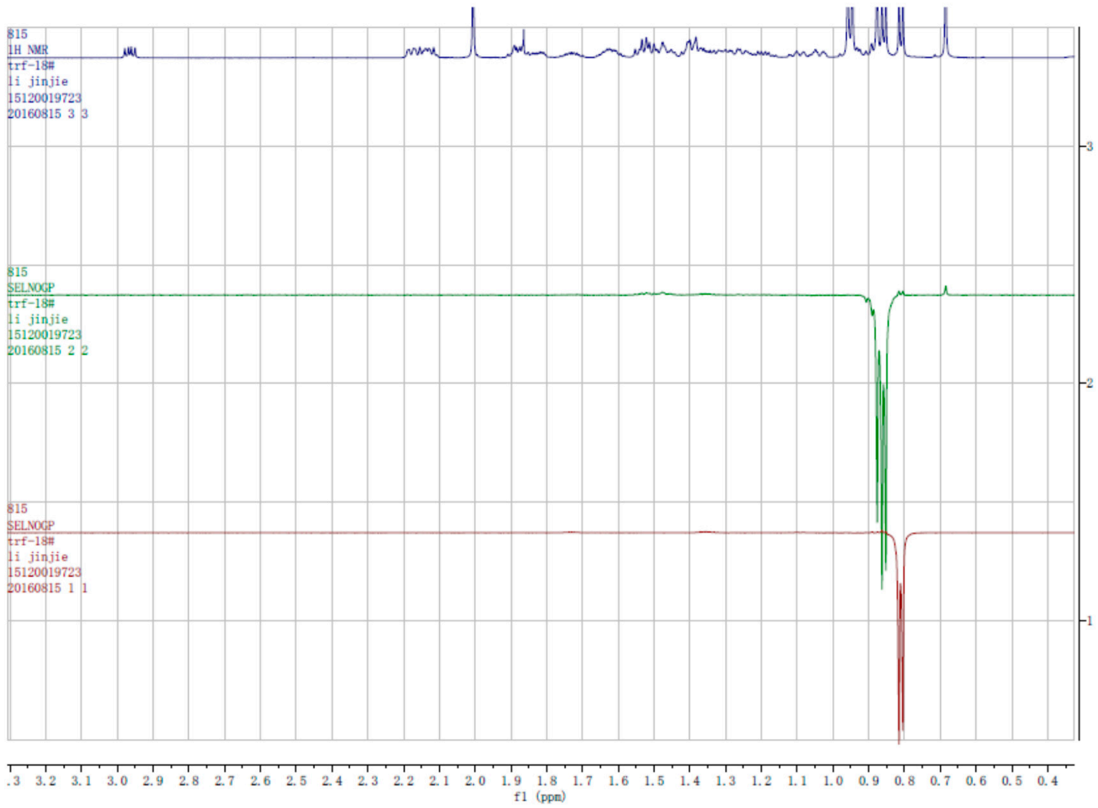

Figure S12. The NOE-3 Spectrum of Compound **1** in Pyr.

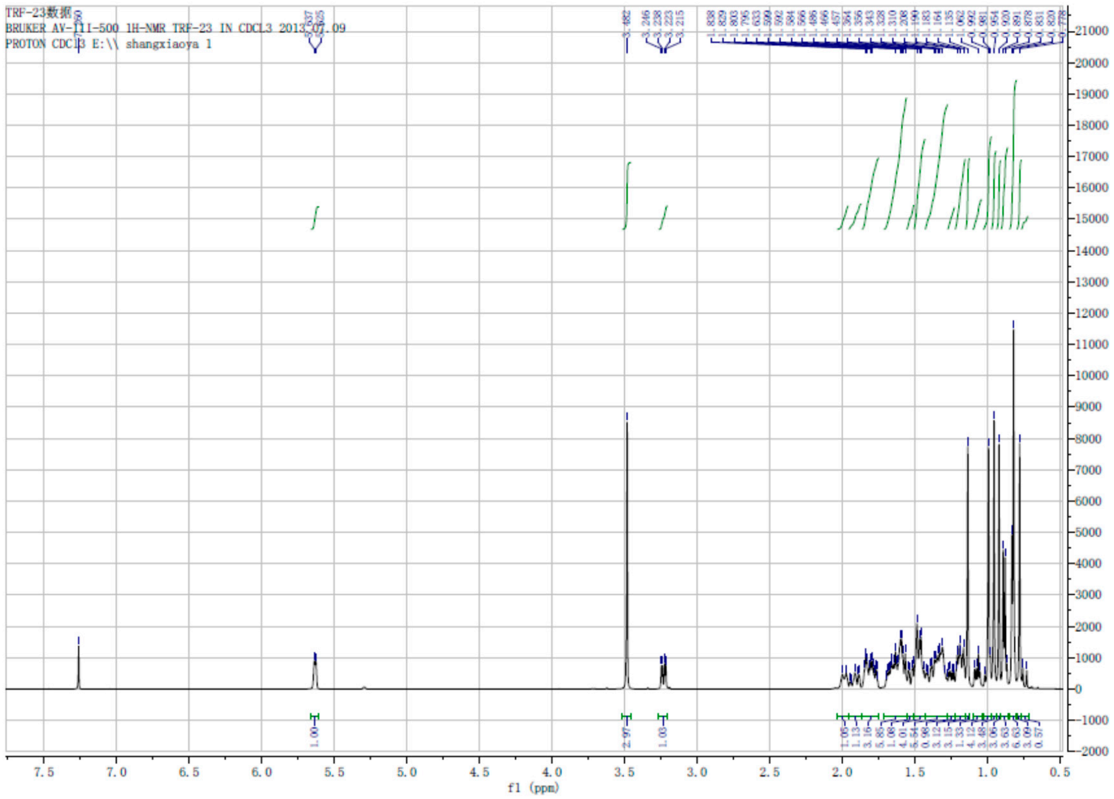

Figure S13. The ^{1}H-NMR Spectrum of Compound **2** in $CDCl_3$.

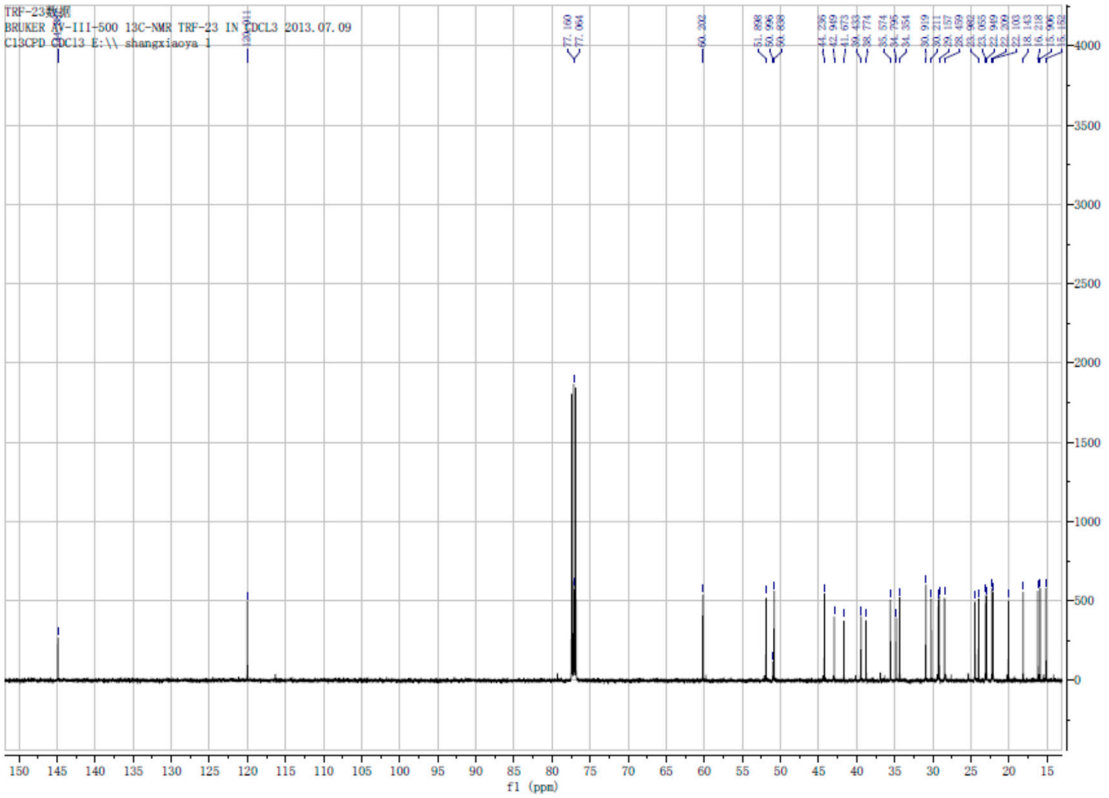

Figure S14. The ^{13}C-NMR Spectrum of Compound **2** in $CDCl_3$.

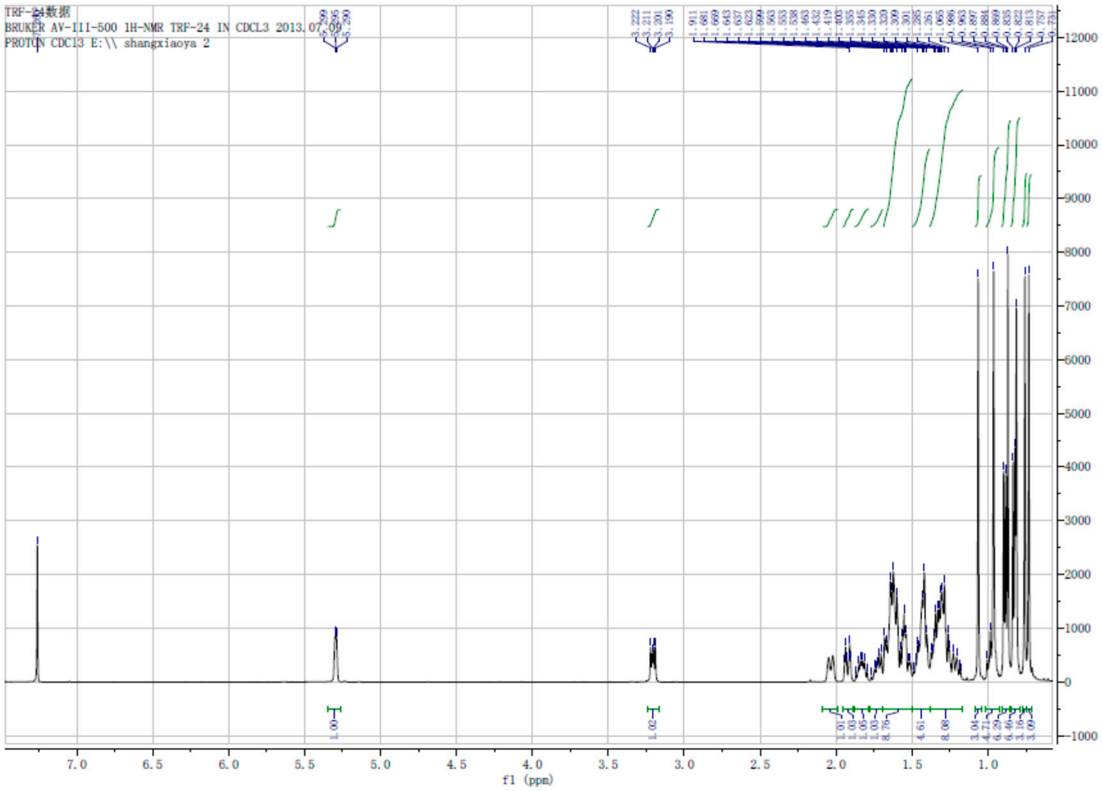

Figure S15. The ^{1}H-NMR Spectrum of Compound **3** in $CDCl_3$.

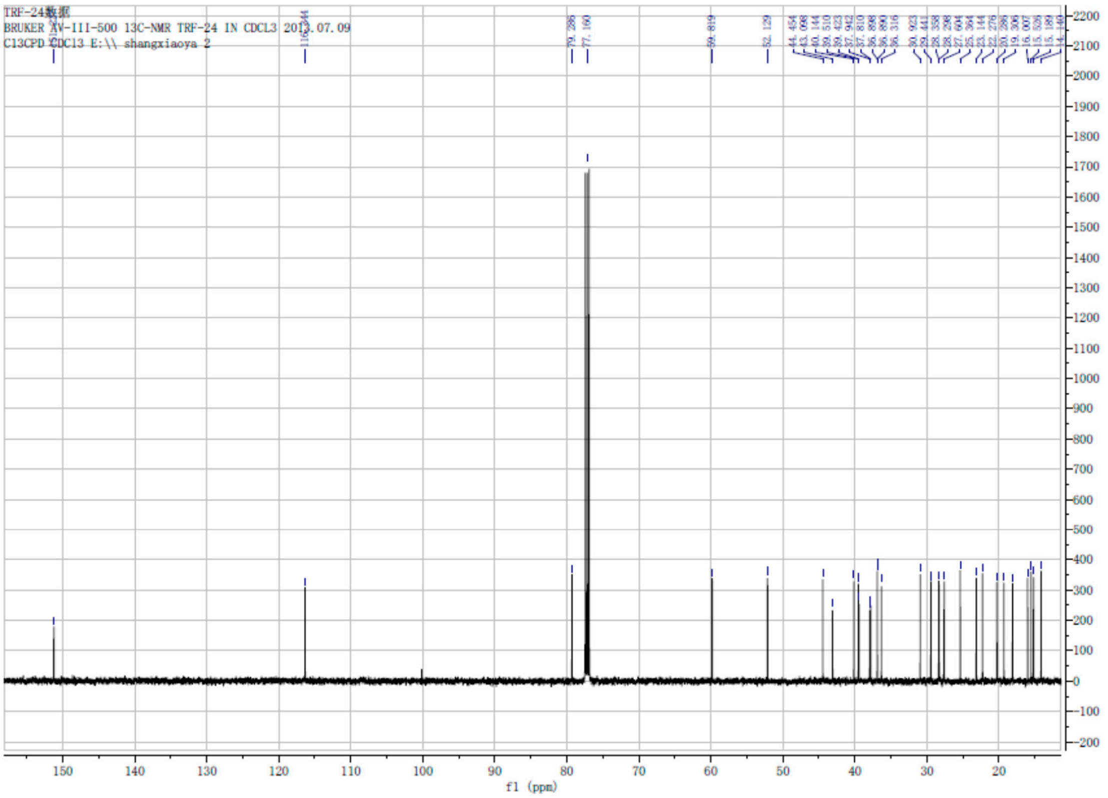

Figure S16. The ^{13}C-NMR Spectrum of Compound **3** in $CDCl_3$.

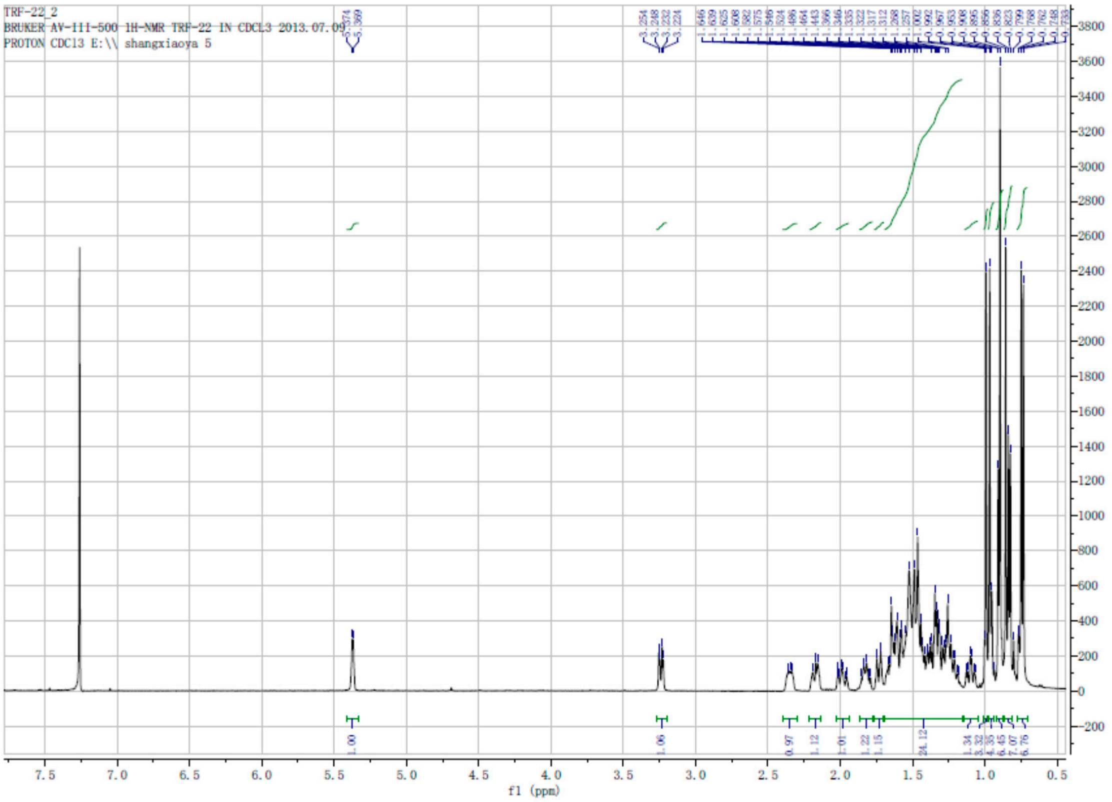

Figure S17. The ^{1}H-NMR Spectrum of Compound **4** in $CDCl_3$.

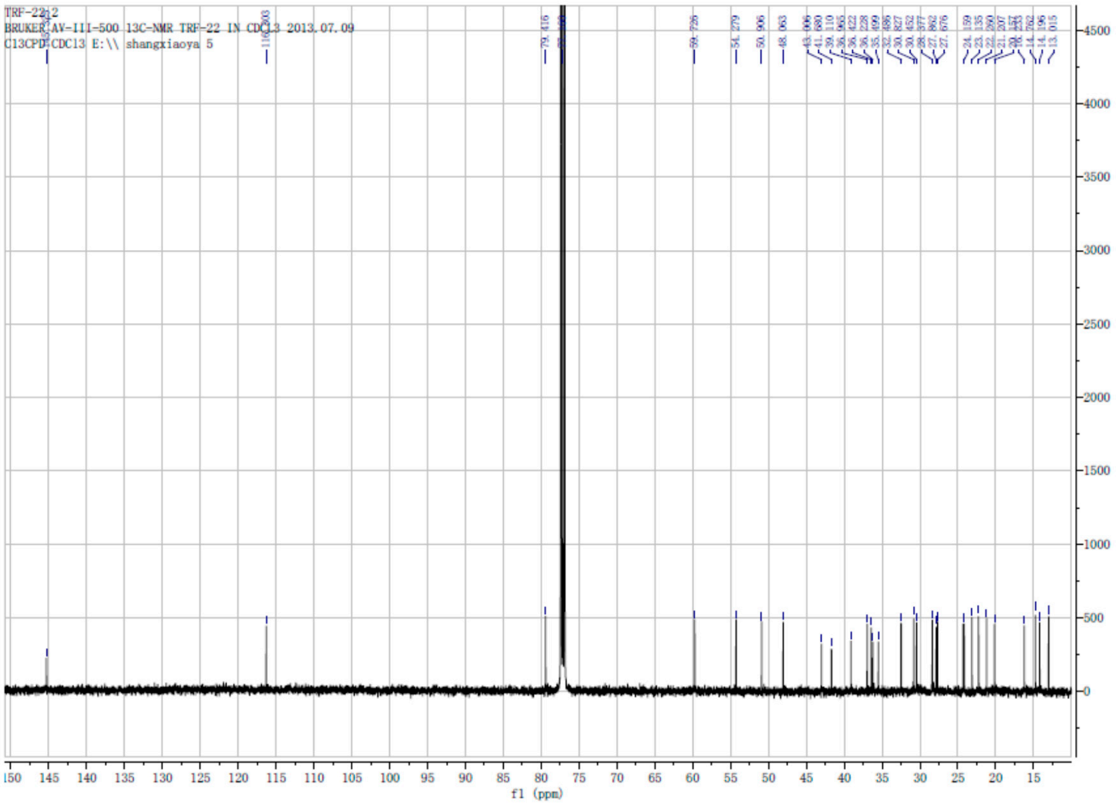

Figure S18. The ^{13}C-NMR Spectrum of Compound **4** in $CDCl_3$.
